# Intraoperative blood transfusion volume is an independent risk factor for postoperative acute kidney injury in type A acute aortic dissection

**DOI:** 10.1186/s12872-020-01727-3

**Published:** 2020-10-14

**Authors:** Yanli Liu, Yuqiang Shang, Ding Long, Li Yu

**Affiliations:** 1 Intensive Care Unit, The Central Hospital of Wuhan, Tongji Medical College, Huazhong University of Science and Technology, Wuhan, Hubei China; 2 Department of Cardiothoracic Surgery, The Central Hospital of Wuhan, Tongji Medical College, Huazhong University of Science and Technology, Wuhan, Hubei China

**Keywords:** Blood transfusion, Acute kidney injury, Type A acute aortic dissection

## Abstract

**Background:**

Type A acute aortic dissection is a life-threatening disease associated with adverse clinical outcomes. Acute kidney injury (AKI) is common after surgery. However, the relationship between intraoperative blood transfusion and postoperative AKI remains unclear.

**Methods:**

The records of 130 patients who underwent type A acute aortic dissection surgery from January 2015 to December 2018 were retrospectively analyzed. According to the Kidney Disease Improving Global Outcomes criteria, postoperative AKI was defined based on serum creatinine concentration. Multivariable logistic regression analysis was applied to estimate the independent association between intraoperative blood transfusion volume and the risk of postoperative AKI.

**Results:**

Postoperative AKI was observed in 82 patients (63.08%). The in-hospital mortality was 16.15% (*n* = 21). Multivariate logistic regression showed that the amount of intraoperative blood transfusion was independently associated with the risk of postoperative AKI in a dose-dependent manner. Every 200 ml increment of blood transfusion volume was associated with a 31% increase in AKI risk (odds ratio 1.31 and 95% confidence interval 1.01–1.71).

**Conclusions:**

Intraoperative transfusion volume may increase the incidence of postoperative AKI. The mechanism and influence of transfusion thresholds on AKI need to be explored in the future.

## Background

Acute aortic dissection remains a clinical emergency characterized by anterior chest or back pain and has high morbidity and mortality, especially for type A acute aortic dissection. Unlike other elective cardiac surgery, in emergency aortic repair for type A acute aortic dissection, patients often suffer from unstable hemodynamics, coagulopathy, and organ malperfusion. Acute kidney injury (AKI) is a common complication and has been reported as an important risk factor for mortality in patients undergoing cardiac surgery [1, 2]. More than 30% of patients undergoing cardiac surgery with cardiopulmonary bypass (CPB) are likely to develop AKI and approximately 3% of patients need renal replacement therapy (RRT) for severe AKI. Various risk factors for AKI, including volume depletion, hypotension, anemia, and blood transfusion, were identified in reports [3, 4]. Freeland and colleagues reported that blood transfusion was an independent predictor of postoperative AKI in patients undergoing coronary artery bypass grafting, aortic or mitral valve surgery, or combined coronary artery bypass grafting and valve surgery [5]. Previous studies have also probed into the relationship between blood transfusion and AKI, suggesting a 10–20% increase in the risk of AKI in patients with cardiac surgery after being given each unit of perioperative blood transfusion [6–10]. Reasonable perioperative management might curb the development of kidney injury [11, 12]. However, the relationship between intraoperative blood transfusion and postoperative AKI in patients undergoing type A acute aortic dissection surgery remains unclear. For this reason, we conducted a retrospective cohort study on patients with type A acute aortic dissection surgery performed with CPB, and further explored the dose–response association between intraoperative blood transfusion volume and postoperative AKI.

## Methods

### Study population

**Fig. 1:**
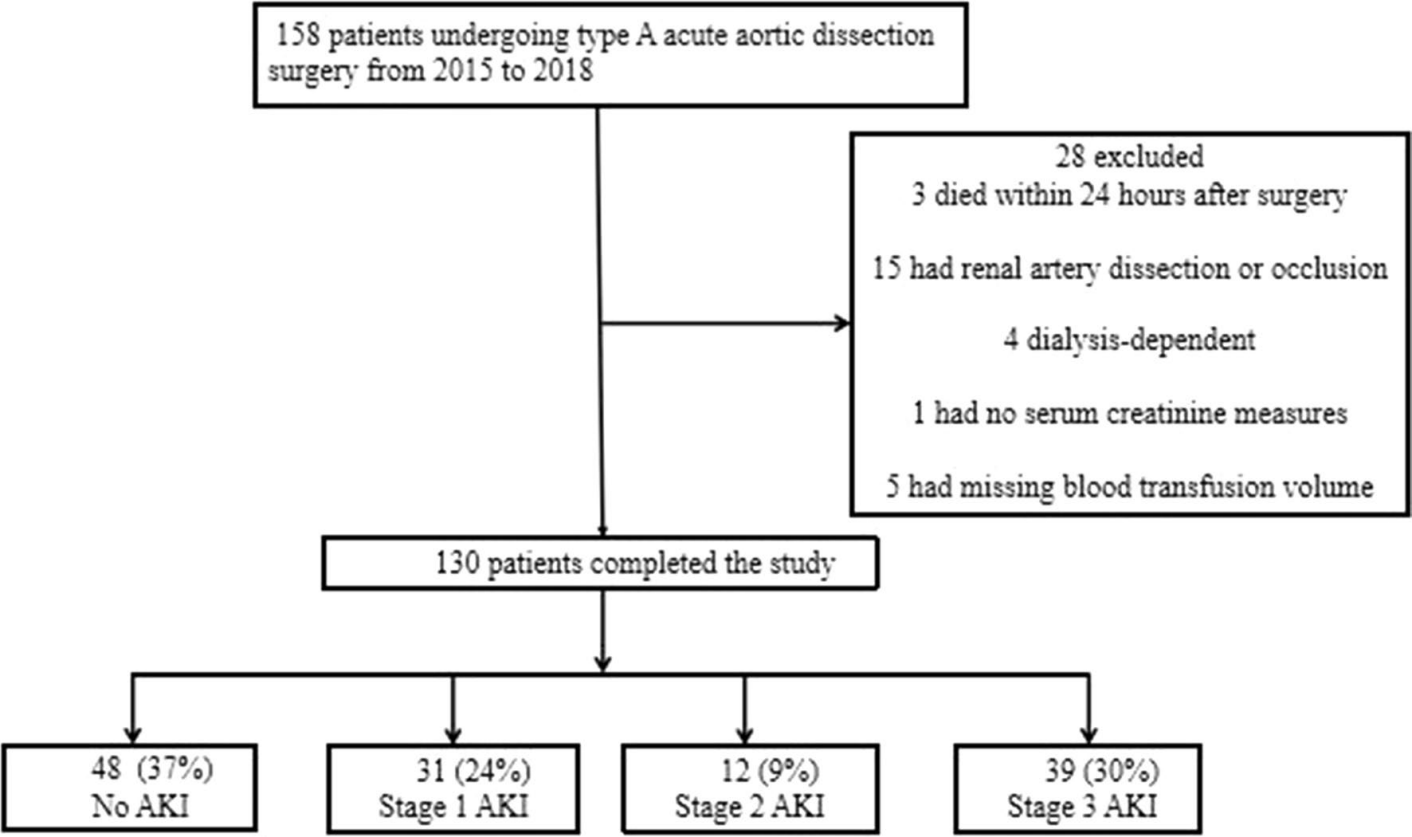
Flowchart of patients underwent type A acute aortic dissection surgery

The study was a retrospective cohort design, including patients who underwent type A acute aortic dissection surgery in the Center Hospital of Wuhan from January 2015 to December 2018. The diagnosis of type A acute aortic dissection was in accordance with contrast-enhanced computed tomography. This study was approved by the ethics committees of this hospital (Ethics approval NO: 2020.163). Data were anonymous, and the requirement for informed consent was waived. The following patients were not eligible for participation: death within 24 h after surgery; renal artery dissection or occlusion; preoperative shock (a systolic blood pressure < 90 mmHg); dialysis-dependent before surgery; no serum creatinine measures after surgery; missing blood transfusion volume. Finally, a total of 130 patients among 158 participants were eligible for the study (Fig. 1).

### Outcome variables

AKI was categorized in accordance with the Kidney Disease: Improving Global Outcomes criteria. Postoperative AKI was defined by the changes in serum creatinine within 48 h after surgery and classified as stage 1, stage 2, and stage 3 on the basis of postoperative serum creatinine. Stage 1 AKI showed more than 50% increase or 0.3 mg/dl increase in serum creatinine. Stage 2 AKI showed 100% increase in serum creatinine. Stage 3 AKI showed 200% or higher or 4 mg/dl increase in serum creatinine or initiation of renal replacement therapy [13]. The last serum creatinine value before surgery was defined as the baseline serum creatinine value. The indications for RRT included acidosis, electrolyte disturbance and fluid overload. The initiation timing of RRT was determined by the clinician. Our primary endpoint was the occurrence of AKI within 48 h after surgery. Secondary endpoints were stages 2–3 AKI within 48 h after surgery, prolonged ventilation, length of stay in the ICU, incidence of renal replacement therapy, adult respiratory distress syndrome (ARDS), perioperative myocardial infarction, stroke, redo surgery, and in-hospital death.

### Surgical technique

All participants underwent right axillary artery cannulation for CPB. CPB was initiated after systemic heparinization maintaining an activated clotting time longer than 480 s. Cooling was initiated after CPB was established. The ascending aorta was clamped and cold blood cardioplegia was infused through the coronary ostia to accomplish cardiac arrest. During CPB, the mean arterial pressure was maintained between 50 and 70 mmHg. An arterial blood gas monitoring was performed every 30 min and the required hemoglobin level was at least 80 g/L. Whether to replace an aortic valve depended on the condition of the aortic valve. If there was moderate or severe aortic regurgitation, Bentall procedure was performed. If the severity of aortic regurgitation was mild, only ascending aorta replacement was performed. For patients with aortic arch involvement in the dissection, total arch replacement combined with stented elephant trunk implantation or hybrid operation was performed. Moderate hypothermia circulatory arrest or deep hypothermia circulatory arrest and selective cerebral perfusion were necessitated in this procedure. Moderate hypothermia was defined as nasopharyngeal temperature between 22 and 25 °C, while deep hypothermia was defined as nasopharyngeal temperature between 18 and 21 °C. After selective cerebral perfusion was terminated, rewarming was initiated. Types of intraoperative blood transfusion included erythrocytes, fresh frozen plasma, platelets and cryoprecipitate. The blood transfusion volume depended on intraoperative bleeding and hemoglobin concentration during CPB.

### Clinical data

The demographic characteristics were collected during patient hospitalization from the electronic medical record system. Information of preoperative hemoglobin, eGFR, preoperative and postoperative serum creatinine levels, type of surgical procedure, time on bypass, aortic cross clamp time, circulatory arrest time, the amount and type of intraoperative blood transfusion, reoperation and other events after surgery were also collected.

### Statistical analysis

The parameters were summarized as mean ± standard deviation (SD) for normally distributed continuous variables, median with interquartile range for continuous variables with skewed distribution, and frequency (percentage) for categorical variables. Multivariable logistic regression was applied to estimate the risk of postoperative AKI. The variables used in multivariable analysis included clinical factors that are known to increase postoperative AKI to control confounding bias. Nonlinear relationship was explored between intraoperative blood transfusion volume and AKI risk following emergent thoracic aortic surgery via the smoothing plot generated by generalized additive model with an adjustment for potential confounders. All of the analyses were performed using R (https://www.R-project.org, version 3.5.2) and EmpowerStats (https://www.empowerstats.com, X&Y Solutions, Inc., Boston, MA) with a two-sided significance threshold of *P* < 0.05.

## Results

### Baseline characteristics

A total of 130 patients with type A acute aortic dissection were included in this study (Table 1) with average age 54.74 ± 11.84 years old. 101 patients (77.69%) were males. The major comorbidities included hypertension (62.31%), diabetes (6.15%), coronary artery disease (5.38%), chronic obstructive pulmonary disease (4.62%), and stroke (6.15%). CPB time was 193 (154–233) min, aortic cross clamp time was 101 (81–124) min, and circulatory arrest time was 16 (0–24) min. 82 patients (63.08%) developed AKI, 31 patients (23.85%) on stage 1 AKI, and 51 patients (39.23%) on stage 2 or 3 AKI.

**Table 1:** Demographic characteristics of patients at baseline

Characteristics	All patients (n = 130)
Age (years), mean (SD)	54.74 (11.84)
Gender, n (%)	
Male	101 (77.69)
Female	29 (22.31)
Hypertension, n (%)	81 (62.31)
Diabetes mellitus, n (%)	8 (6.15)
COPD, n (%)	6 (4.62)
Stroke, n (%)	8 (6.15)
Coronary artery disease, n (%)	7 (5.38)
SBP (mmHg), mean (SD)	133.63 (28.41)
DBP (mmHg), mean (SD)	72.92 (19.31)
eGFR [ml/(min.1.73m^2^)], mean (SD)	91.86 (40.27)
Preoperative hemoglobin (g/l), mean (SD)	122.96 (21.58)
Emergency operation, n (%)	88 (67.69)
CPB time (min), median (IQR)	193 (154–233)
Aortic cross clamp time (min), median (IQR)	101 (81–124)
Circulatory arrest time (min), median (IQR)	16 (0–24)
Total arch replacement, n (%)	76 (58.46)
Semi-arch replacement, n (%)	3 (2.31)
Stented elephant trunk, n (%)	72 (55.38)
Bentall procedure, n (%)	55 (42.31)
Ascending aorta replacement, n (%)	73 (56.15)
David procedure, n (%)	2 (1.54)
Intraoperative erythrocytes use (ml), median (IQR)	2300 (1300–3675)
Intraoperative fresh frozen plasma use (ml), median (IQR)	900 (550–1775)
Intraoperative platelets use (ml), median (IQR)	1200 (600–1200)
Intraoperative cryoprecipitate use (ml), median (IQR)	500 (213–519)
Intraoperative blood transfusion (ml), median (IQR)	4975 (2756–6819)
Postoperative AKI, n (%)	82 (63.08)
Length of ICU (day)	5.0 (3.0–10.0)
Length of mechanical ventilation (day)	2.5 (1.0–6.0)
In-hospital mortality, n (%)	21 (16.15)

AKI, acute kidney injury; COPD, chronic obstructive pulmonary; CPB, cardiopulmonary bypass; DBP, diastolic blood pressure; eGFR, estimated glomerular filtration rate; ICU, intensive care unit; SD, standard deviation; SBP, systolic blood pressure

Demographic and perioperative characteristics stratified according to Improving Global Outcomes criteria [13] were shown in Table 2. The type of procedure, pump time, and the incidence of blood transfusion were associated with AKI development after surgery. No significant difference was found between patients with AKI and those without AKI in terms of age, sex, diabetes, and Circulatory arrest time.

**Table 2:** Comparison of demographic, preoperative, intraoperative, and postoperative characteristics between patients with AKI and those without AKI

	No AKI	Stage 1 AKI	Stage 2 or 3 AKI	*P* value
*N*	48	31	51	–
Age (years), mean (SD)	53.15 (11.61)	57.13 (11.22)	54.78 (12.39)	0.35
Male, n (%)	33 (68.75%)	27 (87.10%)	41 (80.39%)	0.16
Hypertension, n (%)	22 (45.83%)	25 (80.65%)	34 (66.67%)	0.006
Diabetes mellitus, n (%)	1 (2.08%)	3 (9.68%)	4 (7.84%)	0.37
COPD, n (%)	2 (4.17%)	1 (3.23%)	3 (5.88%)	1.00
Stroke, n (%)	3 (6.25%)	2 (6.45%)	3 (5.88%)	1.00
Coronary artery disease, n (%)	1 (2.08%)	4 (12.90%)	2 (3.92%)	0.12
SBP (mmHg), mean (SD)	131.90 (22.70)	125.39 (27.54)	140.27 (32.43)	0.06
DBP (mmHg), mean (SD)	71.02 (16.53)	66.71 (21.57)	78.49 (19.15)	0.018
eGFR [ml/(min.1.73m^2^)], mean (SD)	100.57 (30.65)	71.53 (22.21)	94.84 (51.22)	0.007
Preoperative hemoglobin (g/l), mean (SD)	125.87 (18.39)	120.77 (22.74)	124.53 (22.44)	0.61
Emergency operation, n (%)	21 (43.75%)	25 (80.65%)	42 (82.35%)	< 0.001
CPB time (min), median (IQR)	176 (120–221)	173 (132–212)	210 (185–244)	0.001
Aortic cross clamp time (min), median (IQR)	96 (76–118)	96 (74–107)	120 (95–127)	0.004
Circulatory arrest time (min), median (IQR)	12 (0–20)	15 (0–24)	18 (8–24)	0.26
Nasopharyngeal temperature (°C), mean (SD)	20.9 (2.1)	20.8 (2.1)	20.4 (2.5)	> 0.05
Total arch replacement, n (%)	20 (41.67%)	18 (58.06%)	38 (74.51%)	0.004
Semi-arch replacement, n (%)	2 (4.17%)	1 (3.23%)	0 (0.00%)	0.35
Stented elephant trunk, n (%)	19 (39.58%)	17 (54.84%)	36 (70.59%)	0.008
Bentall procedure, n (%)	27 (56.25%)	14 (45.16%)	14 (27.45%)	0.014
Ascending aorta replacement, n (%)	21 (43.75%)	17 (54.84%)	35 (68.63%)	0.044
Intraoperative erythrocytes use (ml), median (IQR)	1750 (1050–3375)	2200 (1400–2775)	2950 (1950–4350)	0.007
Intraoperative fresh frozen plasma use (ml), median (IQR)	850 (550–1200)	1000 (575–1550)	1000 (550–1925)	0.55
Intraoperative platelets use (ml), median (IQR)	600 (300–1200)	1200 (900–1200)	1200 (750–1200)	0.006
Intraoperative cryoprecipitate use (ml), median (IQR)	350 (0–500)	475 (250–525)	500 (250–560)	0.11
Intraoperative blood transfusion(ml), median (IQR)	2825 (1988–5538)	4400 (2750–5800)	4850 (3650–6875)	0.007
ARDS, n (%)	8 (16.67%)	13 (41.94%)	31 (60.78%)	< 0.001
Perioperative myocardial infarction, n (%)	0 (0.00%)	0 (0.00%)	2 (3.92%)	0.342
Redo surgery, n (%)	2 (4.17%)	3 (9.68%)	13 (25.49%)	0.007
Length of ICU (day), median (IQR)	4.00 (2.75–5.25)	6.00 (3.50–6.50)	9.00 (3.00–14.50)	0.001
Length of mechanical ventilation (day), median (IQR)	2.00 (1.00–3.00)	2.00 (1.00–4.00)	5.00 (2.00–7.50)	< 0.001
In-hospital mortality, n (%)	2 (4.17%)	5 (16.13%)	14 (27.45%)	0.005

AKI, acute kidney injury; ARDS, adult respiratory distress syndrome; COPD, chronic obstructive pulmonary; CPB, cardiopulmonary bypass; DBP, diastolic blood pressure; eGFR, estimated glomerular filtration rate; ICU, intensive care unit; SD, standard deviation; SBP, systolic blood pressure

### In-hospital adverse events and postoperative AKI

Worse in-hospital outcomes were observed in patients with AKI, which contributed to approximately 14.62% of in-hospital death. Patients with AKI had long intubation time and ICU stay, and patients with stage 3 AKI were susceptible to renal replacement therapy. The major adverse events, including perioperative ARDS, perioperative myocardial infarction, stroke, and redo surgery, were more frequent in patients with AKI than those without.

### Blood transfusion and postoperative AKI

**Fig. 2:**
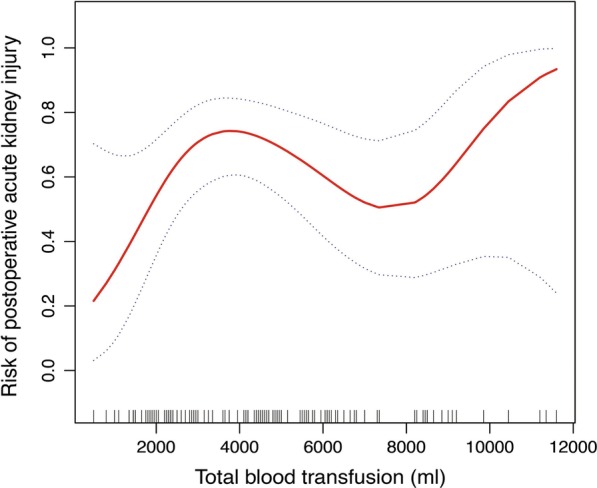
The nonlinear relationship between intraoperative blood transfusion volume and postoperative AKI. The results were generated utilizing generalized additive model and adjusted for age, gender, hypertension, diabetes mellitus, eGFR, emergency operation, CPB time, aortic cross clamp time, nasopharyngeal temperature, total arch replacement, semi-arch replacement, stented elephant trunk, Bentall procedure, ascending aorta replacement, intraoperative erythrocytes use, intraoperative platelets use and redo surgery. The red line indicates the risk of AKI and the blue dot line indicates 95% confidence intervals

No difference was observed in the pre-transfusion Hgb level between patients with and without AKI. Patients with AKI received much more amount of blood transfusion than those without AKI. A nonlinear relationship between intraoperative blood transfusion volume and AKI was explored. We observed the risk of postoperative AKI increased with an increment of blood transfusion volume, and the curve tends to go down when the amount of blood transfusion is above 4000 ml (Fig. 2). In dose–response analyses, every 200 ml increment of blood transfusion volume could lead to a 31% (odds ratio 1.31 and 95% confidence interval 1.01–1.71) increase in AKI risk when the amount of blood transfusion was below 4000 ml. However, null volume association was observed when the amount of blood transfusion was more than 4000 ml (Table 3).

**Table 3:** Multivariable logistic regression analysis to estimate the independent association between blood transfusion volume and the risk of postoperative AKI

Variable	Model 1	*P* value	Model 2	*P* value	Model 3	*P* value
Blood transfusion volume ≤ 4000 ml						
Increase per 200 ml	1.29 (1.08, 1.53)	0.004	1.30 (1.07, 1.57)	0.008	1.31 (1.01, 1.71)	0.044
Blood transfusion volume > 4000 ml						
Increase per 200 ml	1.02 (0.96, 1.07)	0.57	1.01 (0.96, 1.07)	0.70	1.02 (0.96, 1.08)	0.61

Model 1: Crude model

Model 2: Adjusted for age, gender, hypertension, and diabetes mellitus

Model 3: Additional adjust for eGFR, emergency operation, CPB time, aortic cross clamp time, nasopharyngeal temperature, total arch replacement, semi-arch replacement, stented elephant trunk, Bentall procedure, ascending aorta replacement, intraoperative erythrocytes use, intraoperative platelets use and redo surgery

## Discussion

Type A acute aortic dissection is a severe disease related to increased morbidity and mortality. Patients undergoing cardiac surgery suffer from a series of complications, including bleeding, ARDS, acute myocardial infarction, stroke, AKI, and infection. Postoperative AKI is a common complication of cardiac surgery with CBP. Volume depletion, hypotension, anemia, and blood transfusion increase the incidence of AKI [3, 4]. In this study, We explored the relationship between intraoperative blood transfusion volume and postoperative AKI for patients with type A acute aortic dissection surgery and found that intraoperative blood transfusion volume was an independent risk factor for postoperative AKI. Over the last decade, the risk of blood transfusion for AKI has been the focus of many observational studies. Freeland found that patients with postoperative AKI seemed to have a high incidence of blood transfusion [5]. Koch et al*.* reported that perioperative red blood cell transfusion was the independent predictor of postoperative AKI in patients undergoing isolated coronary artery bypass grafting [7]. Meanwhile, blood transfusion was strongly associated with AKI in a retrospective cohort study of patients with acute coronary syndrome undergoing PCI, which was consistent with our study to some extent [3]. However, some studies reported null association between blood transfusion and postoperative AKI, presumably due to the limited sample size and heterogeneous patients’ characteristics [14, 15].

To the best of our knowledge, the current study is the first to investigate the association of intraoperative blood transfusion volume with postoperative AKI in patients undergoing type A acute aortic dissection surgery. We observed a nonlinear relationship between intraoperative blood transfusion volume and postoperative AKI. A 31% increment of the postoperative AKI risk was observed for every 200 ml of blood transfusion received when the total volume was lower than 4000 ml. However, no difference was observed when the blood transfusion volume was higher than 4000 ml. Different from the nonlinear association reported in our study, Karkouti et al*.* found a linear relationship between blood transfusion volume and risk of AKI in a randomized goal-directed fluid resuscitation study [10]. In their study, non-cardiac surgery patients were included and the amount of blood transfusion was relatively small, which might account for the difference.

AKI is a common adverse event for type A acute aortic dissection. Our study also evaluated the relationship between postoperative AKI and in-hospital major adverse events. An association was found between the incidence of AKI and major adverse events. Patients with AKI were associated with long ventilation time and prolonged ICU stay. Moreover, the severity of AKI was correlated with worse in-hospital outcomes. These associations were confirmed in prior studies [16, 17].

The mechanisms through which blood transfusion volume increased the risk of postoperative AKI are not illuminated, but several potential explanations are considered. The pathogenesis of AKI is mostly related to inflammation, renal hypoxia, impairment of tissue oxygen delivery, and oxidative stress. Patients who undergo cardiac surgery with CPB are subjected to the initiation phase of ischemia–reperfusion kidney injury and the extension phase of kidney injury. The initiation phase is characterized by renal artery vasoconstriction and increased oxygen consumption. Patients in the initiation phase have an increased risk of aggravation to the extension phase, which may lead to AKI development if they cannot recover or if they suffer from other external risk factors, such as blood transfusion, ischemia, and anemia [10, 18, 19]. Red blood cell transfusion is common during cardiac surgery with CPB. Red blood cells during storage undergo a series of changes, including decreased deformability, increased fragility, progressive hemolysis, and accumulation of free hemoglobin and iron that may accelerate organ tissue dysfunction [20–22]. Macrophages under normal circumstances dispose of red blood corpuscles and release iron to the circulation bound to transferrin. However, a certain amount of free hemoglobin and iron released by macrophages fails to bind to the iron binding sites on the iron-carrier protein transferrin in circulation after blood transfusion and is toxic to the kidneys [10, 23]. Also, dysregulation of renal haemodynamics is typical of AKI. Platelets play a crucial part in renal haemodynamic processes by regulating the endothelial vascular permeability. Activation of platelets could release granules and microvesicles, which are associated with the pathophysiology of AKI [24, 25]. Other evidence further links blood transfusion and AKI. Patients with persistent positive fluid balance are likely inclined to suffer from kidney injury. Increased kidney interstitial pressure and kidney venous pressure due to the excess fluid that overwhelms the limited capacity of the kidneys lead to the decrease in glomerular filtration rate. Fluid accumulation in patients may be associated with increased mortality. Fluid overload causes visceral and peripheral edema, which leads to difficult organic functioning and delays the AKI diagnosis due to the dilution of serum creatinine [26, 27]. A randomized trial concluded that goal-directed therapy decreased blood transfusion and the incidence of AKI attributing to this effect to improved fluid management [28]. Thus, the important risk factors may be interrelated to AKI development.

### Study limitations

This study is subjected to several limitations. Firstly, the causality could not be established because of the nature of the observational study. Thus, further prospective intervention study is needed. Secondly, type A acute aortic dissection surgery is a complicated procedure. We are often forced into the situation where multiple transfusions are required. The appropriate timing of blood transfusion could not be determined, which is an interesting topic to study in the future. Thirdly, because the sample size for this study is relative small, perhaps the influence of blood transfusion volume becomes less important. Fourthly, multiple factors may cause postoperative AKI after aortic surgery, including preoperative shock or hypotension, renal artery dissection or occlusion, anemia, surgical trauma, prolonged use of CPB, hypothermia, and extensive blood product, during the study period, it is very difficult to take all factors into consideration. Fortunately, most important variables are included in our study to discuss the incidence of AKI. Fifthly, the residual confounding and bias could not be totally addressed in the observational study.

## Conclusions

Our study showed that the amount of intraoperative blood transfusion was an independent risk factor for postoperative AKI in patients with type A acute aortic dissection. Intraoperative transfusion volume might increase the incidence of postoperative AKI, and the mechanism underlying transfusion thresholds on AKI should be further investigated.

## Data Availability

The datasets used and/or analysed during the current study are available from the corresponding author on reasonable request.

## Abbreviations

AKI: Acute kidney injury
ARDS: Adult respiratory distress syndrome
COPD: Chronic obstructive pulmonary
CPB: Cardiopulmonary bypass
DBP: Diastolic blood pressure
eGFR: Estimated glomerular filtration rate
ICU: Intensive care unit
RRT: Renal replacement therapy
SD: Standard deviation
SBP: Systolic blood pressure
